# Artefacts in 1.5 Tesla and 3 Tesla cardiovascular magnetic resonance imaging in patients with leadless cardiac pacemakers

**DOI:** 10.1186/s12968-018-0469-4

**Published:** 2018-07-05

**Authors:** Daniel Kiblboeck, Christian Reiter, Juergen Kammler, Pierre Schmit, Hermann Blessberger, Joerg Kellermair, Franz Fellner, Clemens Steinwender

**Affiliations:** 1 Department of Cardiology, Kepler University Hospital Linz, Med Campus III, Krankenhausstraße 9, 4021 Linz, Austria; 2 Department of Radiology, Kepler University Hospital Linz, Linz, Austria; 3 Medical Faculty of the Friedrich Alexander University of Erlangen-Nürnberg, Erlangen, Germany; 4 Department of Internal Medicine II, Paracelsus Medical University Salzburg, Salzburg, Austria

**Keywords:** Artefacts, Leadless cardiac pacemakers, Cardiovascular magnetic resonance imaging, 1.5 Tesla, 3 Tesla

## Abstract

**Background:**

There are limited data on patients with leadless cardiac pacemakers (LCP) undergoing magnetic resonance imaging. The aim of this prospective, single-center, observational study was to evaluate artefacts on cardiovascular magnetic resonance (CMR) images in patients with LCP.

**Methods:**

Fifteen patients with Micra™ LCP, implanted at least 6 weeks prior to CMR scan, were enrolled and underwent either 1.5 Tesla or 3 Tesla CMR imaging. Artefacts were categorized into grade 1 (excellent image quality), grade 2 (good), grade 3 (poor) and grade 4 (non-diagnostic) for each myocardial segment. One patient was excluded because of an incomplete CMR investigation due to claustrophobia.

**Results:**

LCP caused an arc-shaped artefact (0.99 ± 0.16 cm^2^) at the right ventricular (RV) apex. Of 224 analyzed myocardial segments of the left ventricle (LV) 158 (70.5%) were affected by grade 1, 27 (12.1%) by grade 2, 17 (7.6%) by grade 3 and 22 (9.8%) by grade 4 artefacts. The artefact burden of grade 3 and 4 artefacts was significantly higher in the 3 Tesla group (3 Tesla vs 1.5 Tesla: 3.7 ± 1.6 vs 1.9 ± 1.4 myocardial segments per patient, *p* = 0.03). A high artefact burden was particularly observed in the mid anteroseptal, inferoseptal and apical septal myocardial segments of the LV and in the mid and apical segments of the RV. Quantification of LV function and assessment of valves were feasible in all patients. We did not observe any clinical or device-related adverse events.

**Conclusion:**

CMR imaging in patients with LCP is feasible with excellent to good image quality in the majority of LV segments. The artefact burden is comparable small allowing an accurate evaluation of LV function, cardiac structures and valves. However, artefacts in the mid anteroseptal, inferoseptal and apical septal myocardial segments of the LV due to the LCP may impair or even exclude diagnostic evaluation of these segments. Artefacts on CMR images may be reduced by the use of 1.5 Tesla CMR scanners.

## Background

Cardiovascular magnetic resonance (CMR) imaging, which has become a versatile, non-invasive imaging tool, allows a comprehensive evaluation of patients with cardiovascular diseases [1]. The different CMR imaging sequences offer the assessment of myocardial function, wall motion abnormalities, viability, coronary perfusion, valves and tissue characterization [1]. Potential hazards for patients with conventional cardiac pacemakers undergoing magnetic resonance imaging (MRI) are radiofrequency-induced heating of lead tips, pacing dysfunction and changes in capture threshold [2]. Several studies have demonstrated safety and feasibility of MRI conditional cardiac pacemakers and implantable cardioverter defibrillators (ICD) [3–11]. Leadless cardiac pacemaker (LCP) therapy was recently introduced clinically to overcome complications in transvenous pacemaker therapy, such as lead dislogdement and perforation with pericardial effusion, pocket hematoma and device infections [12–14]. The Micra™ LCP (Medtronic, Minneapolis, USA), which was investigated in this study, is a MRI conditional cardiac, single chamber pacemaker. The device sizes of 25.9 × 6.7 mm with an integrated lithium silver vanadium oxide, carbon monofluoride battery covered in titanium and is fixed with self-expanding nitinol tines in the right ventricle (RV) [14].

Pacemakers cause metallic susceptibility artefacts due to distortion of the magnetic field [15]. There are limited data about patients with LCP undergoing CMR imaging [16]. To the best of our knowledge, there are no prospective studies in the literature about artefacts on CMR imaging in patients with implanted LCP. It is unknown, whether CMR imaging provides best image quality or less artefacts using 1.5 or 3 Tesla CMR scanners in LCP patients.

## Methods

Fifteen patients with an LCP (Micra™, Medtronic, Minneapolis, USA) implanted at least 6 weeks prior to CMR scan were enrolled in this prospective, single-center, observational study. Patients with other ferromagnetic implanted devices which may interact with the CMR scanner were excluded. The study participants were randomized in a 1:1 ratio into two groups: The study participants underwent CMR imaging in either a 1.5 Tesla CMR scanner (Magnetom Avanto Fit, Siemens Healthineers, Erlangen, Germany) or a 3 Tesla CMR scanner (Magnetom Skyra, Siemens Healthineers) with a maximum gradient field of 45mT/m and a slew rate of 200 T/m/s. The Micra™ LCP is a MRI conditional single-chamber cardiac pacemaker and the device sizes 25.9 × 6.7 mm. LCP were interrogated before and immediately after the CMR scan and were programmed to an asynchronous, MRI conditional pacing mode (VOO, 80 bpm) for the CMR scan. During the CMR scan patients were monitored by continuous electrocardiogram (ECG) and pulse oximetry. Blood pressure measurements were performed before and after CMR scans. An intercom was available for patient communication in the CMR scanners.

The CMR protocol was conducted according to the recommendations of the Society for Cardiovascular Magnetic Resonance (SCMR) [17]. We obtained multiple slice transversal balanced steady-state free precession (bSSFP) images for anatomical orientation and bSSFP cine images in the long axis (4- and 2-chamber view of the left ventricle (LV), LV outflow tract view, 2-chamber view of the right ventricle (RV), RV outflow tract view) and multiple short axis of the LV for function evaluation. A fast low angle shot (FLASH) gradient echo (GRE) based sequence was performed of the 4-chamber view of the LV and T1- and T2-weighted Turbo Spin Echo (TSE) sequences were obtained of the 4-chamber view and in the short axis. CMR sequences and parameters for 1.5 and 3 Tesla CMR scans are shown in Table 1. One patient was excluded from the analysis because of an incomplete CMR investigation due to claustrophobia.

**Table 1:** CMR scan protocols at 3 Tesla and 1.5 Tesla

**3 T**	**TF single-shot localizer**	**TF single-shot localizer TRA**	**TF single-shot localizer 2ch**	**TF single-shot localizer 4ch**	**TF single-shot localizer SA**	**TF frequency-scout 4ch**	**TF cine 4ch**	**FLASH cine 4ch**	**TF cine SAG**	**TF cine 2ch**	**TF cine 2ch RV**	**TF cine LVOT**	**TF cine RVOT**	**T2 TSE DB FS SA**	**T2 TSE DB FS 4ch**	**T1 TSE DB 4ch**	**T1 TSE DB SA**
Time of acquisition [sec]	12	45	1	1	8.6	12	9.5	11	159	9.5	9.5	11	11	9.5	9.5	11	12
Slices/slab	5, 5, 3	30	1	1	9	1	1	1	9	1	1	1	1	1	1	1	1
Slice group	3	1	1	1	1	1	1	1	1	1	1	1	1	1	1	1	1
Total slices	13	30	1	1	9	1	1	1	9	1	1	1	1	1	1	1	1
Distance factor [%]	200	20	20	20	100	20	20	20	67	20	20	20	20	50	50	50	50
Orientation	SAG COR TRA	TRA	2ch	4ch	SA	4ch	4ch	4ch	SA	2ch	2ch RV	LVOT	RVOT	SA	4ch	4ch	SA
Phase encoding	A> > P A> > P R> > L	A> > P	A> > P	A> > P	R> > L	A> > P	A> > P	A> > P	R> > L	A> > P	L> > R	A> > P	A> > P	R> > L	A> > P	A> > P	R> > L
FOV read [mm]	430	380	420	420	420	420	380	380	380	400	400	380	380	400	380	380	400
FOV phase [%]	90	90	100	100	100	81	81	80	84	81	81	100	100	78	78	75	75
Slice thickness [mm]	6	5	6	6	6	6	6	6	6	6	6	6	6	8	8	8	8
TR [ms]	237	297	269	260	260	269	41	50	41	41	41	41	41	700	700	700	700
TE [ms]	1.14	1.2	1.18	1.14	1.14	1.21	1.25	2.43	1.25	1.24	1.24	1.25	1.25	71	71	31	31
Concatenations	13	3	1	1	9	1	1	1	9	1	1	1	1	1	1	1	1
Flip angle	40	40	40	40	40	40	35	12	35	35	35	35	35	180	180	150	150
Matrix	230 × 256 256 × 230	512 × 460	256 × 256	256 × 256	256 × 256	256 × 208	256 × 208	256 × 206	216 × 256	208 × 256	208 × 256	256 × 256	256 × 256	400 × 512	512 × 400	256 × 192	192 × 256
Phase resolution [%]	65	80	65	65	65	66	81	80	81	81	81	81	81	80	80	80	80
Fat suppression	none	none	none	none	none	none	none	none	none	none	none	none	none	FS	FS	none	none
Dimension	2D	2D	2D	2D	2D	2D	2D	2D	2D	2D	2D	2D	2D	2D	2D	2D	2D
Bandwidth [Hz/px]	977	1085	849	977	977	781	1085	543	1085	1085	1085	1085	1085	849	849	781	781
Turbo factor														21	21	9	9
RF pulse type	fast	fast	fast	fast	fast	fast	fast	fast	fast	fast	fast	fast	fast	fast	fast	fast	fast
Gradient mode	fast	fast	fast	fast	fast	fast	fast	fast	fast	fast	fast	fast	fast	normal	normal	fast	fast
**1,5 T**	**TF localizer multi iPAT**	**TF TRA**	**TF 2ch**	**TF 4ch**	**TF SA**	**TF cine 4ch**	**FLASH cine 4ch**	**TF cine SA**	**TF cine 2ch**	**TF cine 2ch RV**	**TF cine LVOT**	**TF cine RVOT**	**T2 TSE DB FS SA**	**T1 TSE DB SA**	**T2 TSE DB FS 4ch**	**T1 TSE DB 4ch**	
Time of acquisition [sec]	16	40	2.4	2.4	12	5.5	6.7	44	6.1	6.1	6.1	6.1	24	13	20	12	
Slices/slab	5, 5, 3	30	1	1	9	1	1	9	1	1	1	1	1	1	1	1	
Slice group	3	1	1	1	1	1	1	1	1	1	1	1	1	1	1	1	
Total slices	13	30	1	1	9	1	1	9	1	1	1	1	1	1	1	1	
Distance factor [%]	200	20	20	20	100	20	20	67	20	20	20	20	20	25	20	25	
Orientation	SAG COR TRA	TRA	2ch	4ch	SA	4ch	4ch	SA	2ch	2ch RV	LVOT	RVOT	SA	SA	4ch	4ch	
Phase encoding	A> > P A> > P R> > L	A> > P	A> > P	A> > P	A> > P	A> > P	R> > L	A> > P	A> > P	A> > P	A> > P	A> > P	A> > P	H> > F	R> > L	R> > L	
FOV read [mm]	400	350	400	400	400	380	380	400	400	400	380	380	400	400	380	380	
FOV phase [%]	100	89.8	100	100	100	83.3	83.3	85.8	99.2	99.2	99.2	99.2	75	75	75	75	
Slice thk [mm]	8	5	8	6	6	6	6	6	6	6	6	6	8	8	8	8	
TR [ms]	288	341	285	288	288	40	51	52	41	41	41	41	700	700	700	700	
TE [ms]	1.1	1.2	1.1	1.1	1.1	1.	2.	1.2	1.2	1.2	1.2	1.2	81	29	81	29	
Concatenations	1	3	1	1	1	1	1	9	1	1	1	1	1	1	1	1	
Flip angle	80	70	80	80	80	70	15	70	70	70	70	70	180	180	180	180	
Matrix	256 × 256	512 × 460	256 × 256	256 × 256	256 × 256	240 × 200	200 × 240	206 × 240	238 × 240	238 × 240	240 × 238	238 × 240	384 × 512	384 × 512	512 × 384	512 × 384	
Phase resolution [%]	66	80	65	65	65	85	85	85	85	85	85	85	80	80	80	80	
Fat suppr.	none	none	none	none	none	none	none	none	none	none	none	none	FS	none	FS	none	
Dimension	2D	2D	2D	2D	2D	2D	2D	2D	2D	2D	2D	2D	2D	2D	2D	2D	
Bandwidth [Hz/px]	1149	977	1149	1149	1149	947	496	947	947	947	947	947	235	305	235	305	
Turbo factor													23	11	23	11	
RF pulse type	fast	fast	fast	fast	fast	fast	fast	fast	fast	fast	fast	fast	fast	fast	fast	fast	
Gradient mode	fast	fast	fast	fast	fast	fast	fast	fast	fast	fast	fast	fast	fast	fast	fast	fast	

*TF* TrueFISP, *FLASH* fast low angle shot, *TSE* turbo spin echo, *DB* dark blood, *FS* fat saturation, *iPAT* integrated parallel acquisition techniques, *TRA* transversal, *SAG* sagittal, *COR* coronal, *2ch* 2 chamber, *4ch* 4 chamber, *LVOT* left ventricle outflow tract, *RV* right ventricle, *RVOT* right ventricle outflow tract, *SA* short axis

All available CMR images were reviewed by four experienced CMR observers (two cardiologists and two radiologists). In case of inter-observer differences in artefact quantification agreement was reached in case discussion. The LV was divided into 16 segments according to the American Heart Association (AHA) 16 myocardial segmentation classification system [18]. The RV was divided into 3 segments (basal, mid and apical).

LCP related artefacts were graduated as suggested by Klein-Wiele et al. [19]:

- Grade 1: excellent image quality, no artefacts affecting myocardial segments or cardiac structures
- Grade 2: good image quality with artefact adjacent to the myocardial segments or cardiac structures, delineation of myocardial borders may be limited, no impact on diagnostic value
- Grade 3: artefact moderately affecting cardiac structures, less than half of the myocardial segment is superimposed by the artefact
- Grade 4: poor image quality with significant artefact affecting more than half of the myocardial segment, non-diagnostic image

The artefact size was measured for every patient on short axis bSSFP cine images which showed the largest artefact. The artefact burden was calculated as affected myocardial segments by grade 1 (excellent), grade 2 (good), grade 3 (moderately) and grade 4 artefacts (poor image quality) divided by 224 (16 myocardial segments × 14 study participants) for the LV and divided by 42 (3 myocardial segments × 14 study participants) for the RV. The artefact burden ratio per patient was calculated as affected myocardial segments by grade 3 and 4 artefacts on 1.5 Tesla and 3 Tesla CMR images for the LV and RV.

The study design was approved by the local ethics committee and was conducted according to the Declaration of Helsinki. Written informed consent was obtained from all study participants.

### Statistical analysis

Categorical parameters are described as absolute number and percentage. Continuous values are presented as means ± standard deviation or means with 95% confidence intervals (95% CI). Differences between groups involving normally distributed data were analyzed by the unpaired t test; those involving not normally distributed data, by the Mann-Whitney U test; and those involving proportions, by the chi-square test. A two-sided *p*-value < 0.05 was considered statistically significant. All calculations were performed with SPSS statistical software (Version 21, SPSS Inc., Chicago, Illinois, USA).

## Results

### Baseline characteristics

Fifteen patients were enrolled in this prospective, single-center, observational study. The 1.5 Tesla and 3 Tesla group comprised seven patients each. One study participant was excluded from the study because of an incomplete CMR investigation due to claustrophobia. The remaining 14 patients (female: *n* = 3) had a mean age of 77.8 ± 14.6 years and all of them had undergone implantation of a LCP at least 6 weeks prior the CMR scan. Indications for pacemaker implantation were bradycardic arrhythmias in permanent atrial fibrillation (*n* = 11) or third degree atrioventricular block (*n* = 3). Baseline characteristics of the study population including comorbidities are shown in Table 2.

**Table 2:** Baseline characteristics including indication for pacemaker implantation and comorbidities

Baseline characteristics (*n* = 14)	
Sex	3 female (21.4%)
Age	77.8 ± 14.6 years
Indication for pacemaker implantation	
Bradycardiac arrhythmia in atrial fibrillation	11 (78.6%)
3rd degree AV-block	3 (21.4%)
Comorbidities	
Atrial fibrillation	12 (85.7%)
Coronary artery disease	6 (42.9%)
Hypertension	8 (57.1%)
Diabetes	3 (21.4%)
Chronic kidney disease	6 (42.9%)
Peripheral artery disease	2 (14.3%)
History of stroke	2 (14.3%)

### Image quality and artefacts

**Fig. 1:**
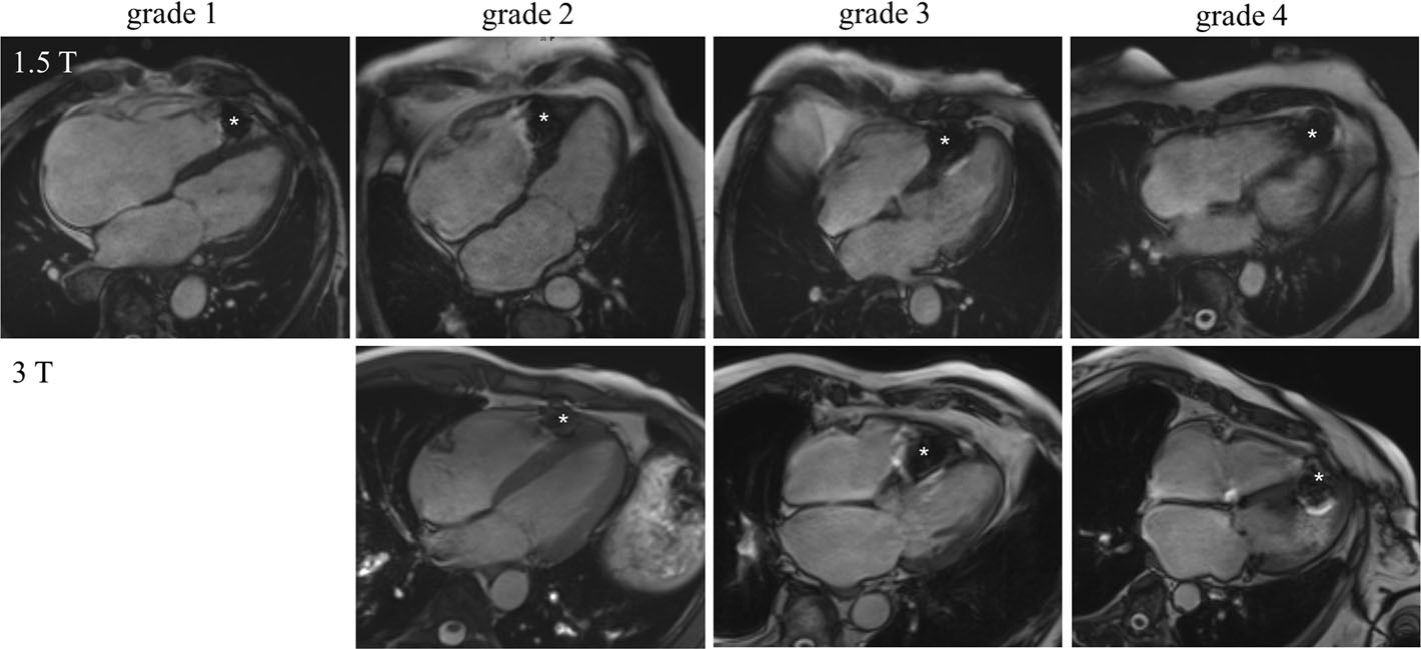
Balanced steady free precession (bSSFP) cine images of the 4 chamber view with grade 1, grade 2, grade 3 and grade 4 artefacts of the apical septal myocardial segment obtained with 1.5 Tesla and 3 Tesla. No patient in the 3 Tesla group showed a grade 1 artefact of the apical septal myocardial segment. * = artefact caused by the leadless cardiac pacemaker (LCP)

**Fig. 2:**
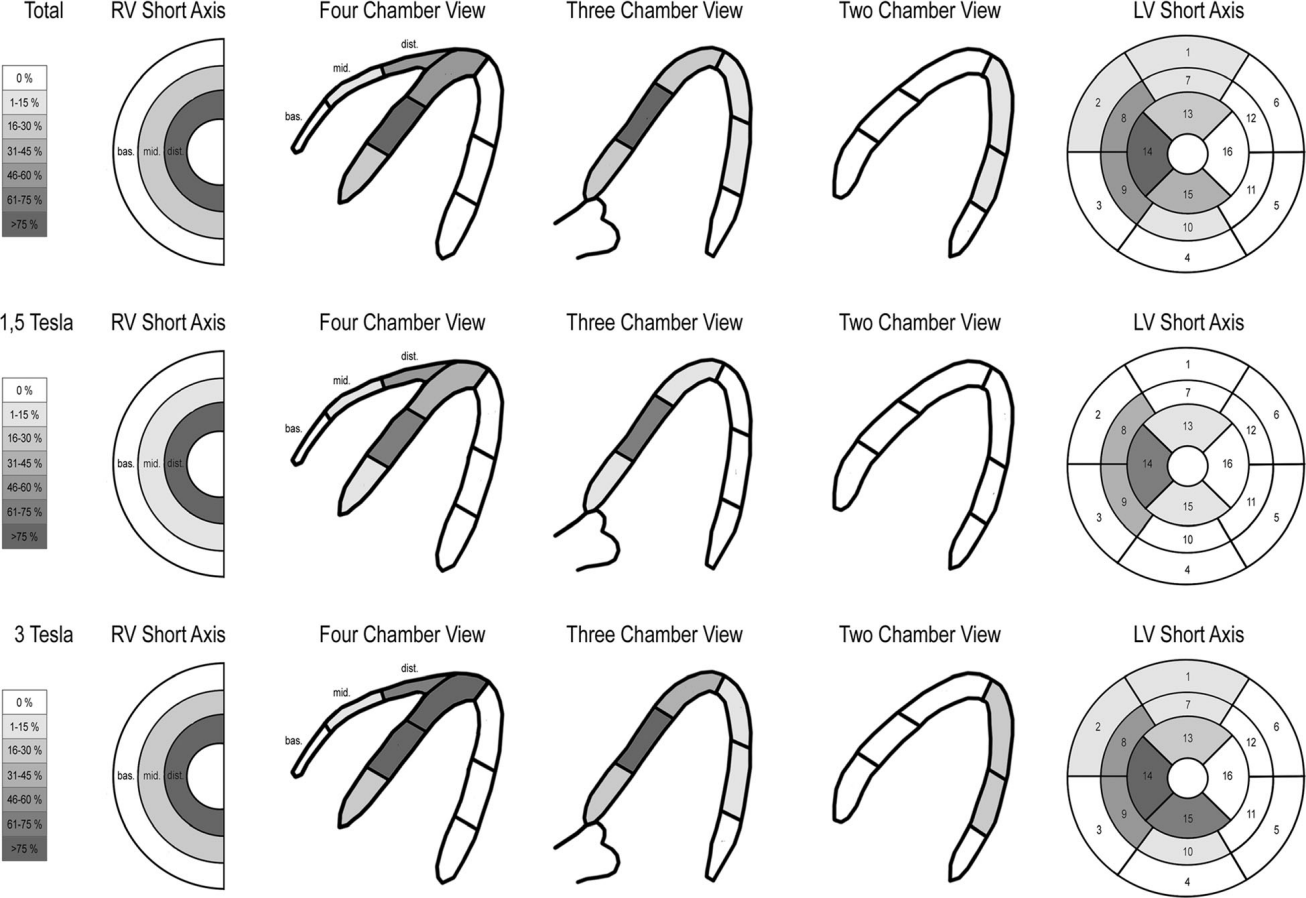
Artefact burden depicting the percentage of grade 3 and 4 artefacts in each myocardial wall segment in the short axis on bSSFP cine imaging sequences. Mid anteroseptal, inferoseptal and apical left ventricular (LV) septal segments (AHA segments 8, 9 and 14) and mid and apical right ventricular (RV) segments were affected particularly by artefacts of the LCP. The artefact burden was significantly higher in the 3 Tesla group (3 Tesla vs 1.5 Tesla: 3.7 ± 1.6 vs 1.9 ± 1.4 myocardial segments per patient, *p* = 0.03)

The LCP caused an arc-shaped artefact at the site of implantation of the RV apex (Fig. 1). Of 224 analyzed myocardial segments of the LV, 158 (70.5%) were affected by grade 1, 27 (12.1%) by grade 2, 17 (7.6%) by grade 3 and 22 (9.8%) by grade 4 artefacts. Of 42 analyzed segments of the RV, 20 (47.6%) were affected by grade 1, 6 (14.3%) by grade 2, 5 (11.9%) by grade 3 and 11 (26.2%) by grade 4 artefacts. Representative bSSFP cine images of the 4-chamber view with artefact grading obtained with 1.5 Tesla and 3 Tesla are shown in Fig. 1. The artefact area, which was quantified on short axis bSSFP cine images, was slightly higher but not significantly different in the 3 Tesla group compared to the 1.5 Tesla group (both groups: 0.99 ± 0.16 cm^2^, 3 Tesla vs 1.5 Tesla: 1.02 ± 0.19 cm^2^ vs 0.95 ± 0.14 cm^2^, *p* = 0.41). The artefact burden ratio per patient of affected myocardial segments by grade 3 and 4 artefacts on the short axis bSSFP cine images of the LV was significantly higher in the 3 Tesla group (3 Tesla vs 1.5 Tesla: 3.7 ± 1.6 vs 1.9 ± 1.4 myocardial segments per patient, *p* = 0.03). The detailed analysis of myocardial segments affected by grade 3 and grade 4 artefacts revealed a high artefact burden particularly in the mid anteroseptal, inferoseptal and apical septal myocardial segments (AHA myocardial segments 8, 9, 14) with an artefact burden of 50, 50 and 85.7% in these regions, respectively. These artefacts were more pronounced in patients undergoing CMR on 3 Tesla CMR scanners (3 Tesla vs 1.5 Tesla: 57.1% vs 42.9%. 57.1% vs 42.9, 100% vs 71.4%). There were no artefacts in the basal inferoseptal, inferior, basal to apical anterolateral and inferolateral myocardial segments (AHA myocardial segments 3, 4, 5, 6, 11, 12 and 16). Details of artefact distribution in the short axis are demonstrated in Fig. 2 and Table 3.

**Table 3:** Artefact burden of myocardial segments of the LV and RV according to the AHA myocardial segmentation system in the short axis (*n* = 14)

All [%]	Basal						Mid						Apical				RV		
Artefact	1	2	3	4	5	6	7	8	9	10	11	12	13	14	15	16	basal	mid	apical
grade 1	92.9	78.6	85.7	100	100	100	64.3	21.4	21.4	78.6	100	100	35.7	7.1	42.9	100	100	42.9	0
grade 2	0	14.3	14.3	0	0	0	28.6	28.6	28.6	14.3	0	0	42.9	7.1	14.3	0	0	35.7	7.1
grade 3	7.1	0	0	0	0	0	7.1	7.1	14.3	7.1	0	0	21.4	14.3	42.9	0	0	14.3	21.4
grade 4	0	7.1	0	0	0	0	0	42.9	35.7	0	0	0	0	71.4	0	0	0	7.1	71.4
3 T	1	2	3	4	5	6	7	8	9	10	11	12	13	14	15	16	basal	mid	apical
grade 1	85.7	71.4	85.7	100	100	100	57.1	0	14.3	57.1	100	100	0	0	14.3	100	100	42.9	0
grade 2	0	14.3	14.3	0	0	0	28.6	42.9	28.6	28.6	0	0	71.4	0	14.3	0	0	28.6	0
grade 3	14.3	0	0	0	0	0	14.3	14.3	14.3	14.3	0	0	28.6	0	71.4	0	0	28.6	0
grade 4	0	14.3	0	0	0	0	0	42.9	42.9	0	0	0	0	100	0	0	0	0	100
1.5 T	1	2	3	4	5	6	7	8	9	10	11	12	13	14	15	16	basal	mid	apical
grade 1	100	85.7	85.7	100	100	100	71.4	42.9	71.4	100	100	100	71.4	14.3	71.4	100	100	42.9	0
grade 2	0	14.3	14.3	0	0	0	28.6	14.3	0	0	0	0	14.3	14.3	14.3	0	0	42.9	14.3
grade 3	0	0	0	0	0	0	0	0	28.6	0	0	0	14.3	28.6	14.3	0	0	0	42.9
grade 4	0	0	0	0	0	0	0	42.9	0	0	0	0	0	42.9	0	0	0	14.3	42.9

Quantification of LV function and volumetry were feasible in 14 patients (100%) with a mean LV ejection fraction of 49 ± 7.4%. We experienced more problems of ECG-triggering in patients undergoing 3 Tesla CMR scans (3 Tesla vs 1.5 Tesla: 3 vs 0 patients). In one 3 Tesla patient, pulse-triggering was necessary for image acquisition. Analysis of the RV was evenly impaired in both cohorts due to artefacts of the LCP severely compromising the image quality of particularly the mid and apical free wall of the RV (grade 3 and 4 artefacts RV basal: 0%, mid: 21.4%, apical: 92.9%). The artefact burden ratio of affected myocardial segments by grade 3 and 4 artefacts on short axis bSSFP cine images of the RV revealed 1.1 ± 0.4 segments per patient. Therefore, an exact quantification of RV function was not possible in both groups and the RV function was only assessed visually (normal: *n* = 7, mildly impaired: *n* = 6, moderately impaired: *n* = 0, severely impaired: *n* = 1).

**Fig. 3:**
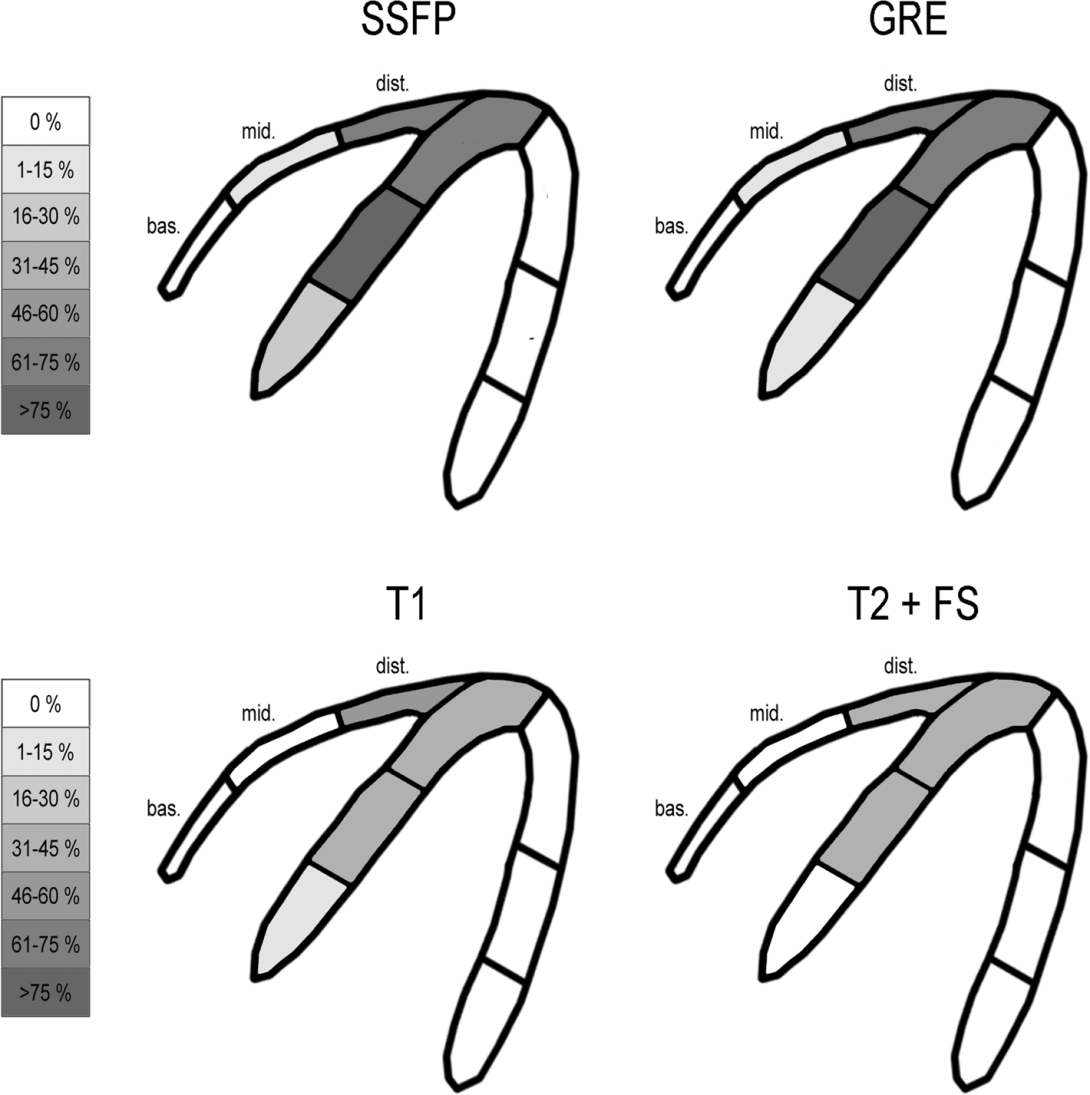
Artefact burden of grade 3 and 4 artefacts of the 4 chamber view with different CMR sequences showed better image quality on T1 and T2 weighted images and only slightly less artefacts on gradient recalled echo (GRE) based fast low angle shot (FLASH) images compared to bSSFP images

**Fig. 4:**
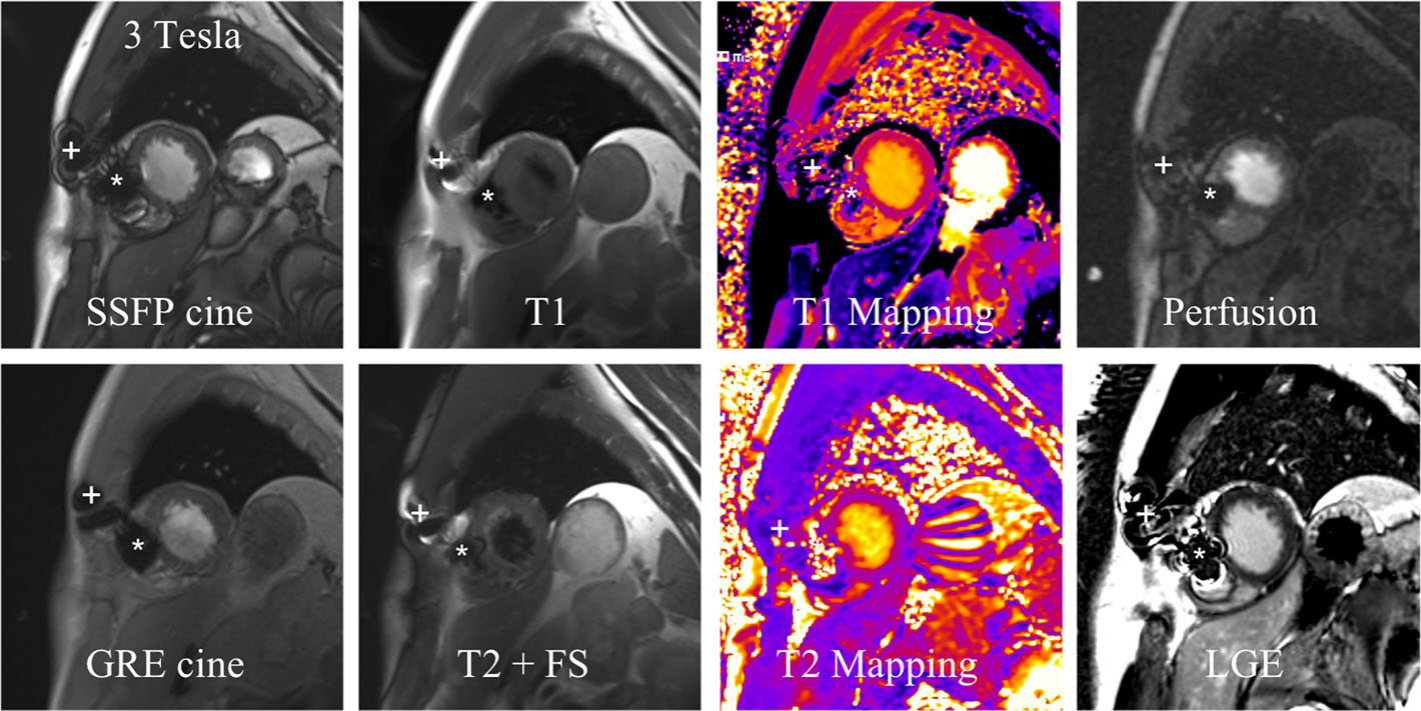
Artefacts due to the LCP in one patient with different CMR sequences obtained with 3 Tesla in short axis orientation showing slightly more artefacts on bSSFP cine images compared to GRE cine, T1, T2 with fat saturation, T1 and T2 mapping, perfusion and LGE images. * = artefact caused by the LCP, + = artefact caused by implantable loop recorder

The evaluation of T1 and T2 weighted images showed better image quality with smaller arc-shaped artefacts compared to bSSFP cine imaging and less grade 3 and 4 artefacts in the mid anteroseptal, inferoseptal and septal apical myocardial segments (Figs. 3 and 4). Furthermore, when comparing 4-chamber view bSSFP cine images and GRE based FLASH cine images, there was only a slightly better image quality on FLASH sequences in the mid and apical septal myocardial segments of the LV (Fig. 3).

Aortic, mitral and tricuspid valves could be assessed in all patients (grade 3 and 4 artefacts: 0, 0, 0%, respectively). We did not observe any hemodynamically relevant tricuspid valve insufficiency in our study cohort of patients with LCP.

### Safety and device integrity

There were no clinical or device-related serious adverse events during CMR scans. The CMR scans, both 1.5 and 3 Tesla, did not lead to malfunction of the implanted LCP devices. We observed no significant change of pacing thresholds (before CMR scan: 0.59 ± 0.15 V/0.24 ms, after: 0.61 ± 0.17 V/0.24 ms, *p* = 0.08) and a marginal, but statistically significant increase of sensing amplitude (before CMR scan: 14.9 ± 4.7 mV, after: 15.8 ± 4.5 mV, *p* = 0.02) and impedances (before CMR scan: 527 ± 100 Ω, after: 541 ± 110 Ω, p = 0.02). Battery voltage remained stable (before CMR scan: > 10 years, after: > 10 years).

## Discussion

This prospective, single-center, observational study, demonstrates that CMR imaging in patients with LCP implanted at least 6 weeks prior to the CMR scan is feasible. Overall image quality was excellent or good in the majority of CMR images (myocardial segments of the LV affected by grade 1: 70.5%, grade 2: 12.1%, grade 3: 7.6%, grade 4: 9.8%). Artefacts occurred particularly in the mid anteroseptal, inferoseptal and septal apical myocardial segments (AHA myocardial segments 8, 9 and 14) and in the mid and apical segments of the RV. 3 Tesla CMR imaging led to a significantly higher artefact burden ratio per patient compared to 1.5 Tesla CMR imaging. Assessment of LV function and aortic, mitral and tricuspid valve, as well as tissue characterization by T1- and T2- weighted imaging was feasible with both modalities.

Several studies have demonstrated the safety and feasibility of MRI conditional, transvenous pacemakers and ICD undergoing MRI [3–11]. Potential adverse effects of MRI on implanted cardiac devices include: radiofrequency-induced heating of the lead tips, pacing inhibition/dysfunction, asynchronous pacing with the possibility of induction of ventricular tachyarrhythmias, change or loss of programmed data and changes in capture treshold [2]. A closer distance of the scanning area to the pacing system and a higher field strength increases this risk [2]. Therefore, pacemakers and ICD including leads must be interrogated before and after MRI scans and have to be programmed to MRI conditional pacing modes during the scan. It is recommended to monitor patients with cardiac devices with ECG, pulse oximetry and blood pressure measurements during MRI scans [2]. Soejima et al. demonstrated in an ex-vivo study the safety for the Micra™ LCP in a not perfused phantom model with a device heating of less than 0.4 °C at 1.5 Tesla and 0.5 °C at 3 Tesla MRI [20]. Furthermore they report no MRI-related complication in a clinical case study. We did not observe any clinical or device-related serious adverse events in our study cohort. Pacing threshold did not change significantly and battery voltage remained stable before and after CMR scans. However, we observed a marginal, but statistically significant increase of sensing amplitude (before CMR scan: 14.9 ± 4.7 mV, after: 15.8 ± 4.5 mV, *p* = 0.02) and impedances (before CMR scan: 527 ± 100 Ω, after: 541 ± 110 Ω, p = 0.02). We do not consider these marginal changes to be clinically relevant because we did not observe any malfunction of LCP during or after CMR scans. Further studies are needed to provide follow up data of LCP after MRI.

Pacemakers and other implanted cardiac electronic devices lead to metallic susceptibility artefacts due to distortion of the magnetic field [15]. Artefacts tend to be larger on 3 Tesla CMR scanners which could be confirmed by our study findings with a higher artefact burden of affected myocardial segments of the LV by grade 3 and 4 artefacts in patients undergoing CMR imaging at 3 Tesla (3 Tesla vs 1.5 Tesla: 3.7 ± 1.6 vs 1.9 ± 1.4 AHA myocardial segments, *p* = 0.03). These susceptibility artefacts were pronounced in particular in the mid anteroseptal, inferoseptal and apical septal AHA myocardial segments 8, 9 and 14 of the LV and were even more pronounced in patients undergoing CMR on 3 Tesla. The RV was mainly affected in the mid and apical myocardial segments. The size of the arc-shaped artefact by the LCP (0.99 ± 0.16 cm^2^) was not significantly different in both groups (3 Tesla vs 1.5 Tesla: 1.02 ± 0.19 cm^2^ vs 0.95 ± 0.14 cm^2^, *p* = 0.41). The size of the area affected by the artefact was 7-fold larger compared to the size of the LCP device itself. Quantification of LV function and volumetry were feasible in all patients (*n* = 14). However, the RV function could only be assessed visually, because of the high artefact burden, demonstrating 7 patients with normal, 6 patients with mildly and 1 patient with severely reduced RV function. However, the impaired RV function cannot be interpreted as a reduced RV function due to the LCP because we did not compare RV function before and after implantation of the LCP.

As described above, mid anteroseptal, inferoseptal and apical septal myocardial segments of the LV and mid and apical segments of the RV were affected by artefacts of the LCP in the majority of patients which may impair or even exclude diagnostic evaluation of these segments especially on perfusion and late gadolinum enhancement images. In contrast to our findings, Klein-Wiele et al. revealed in a population of 61 patients with MRI conditional, transvenous pacemakers no relevant artefacts in patients with right-sided devices irrespective of the imaging sequence [19]. There were no pacemaker induced artefacts in left-sided implants in first pass perfusion sequence, flow analysis and T1 weighted imaging. bSSFP cine sequences tend to have more artefact burden than late gadolinum enhancement sequences [19]. As reported by Sasaki et al. right-sided pacemakers and ICD did not cause susceptibility artefacts on CMR images but artefacts of the anterior and apical LV were described with left-sided ICD [21]. To the best of our knowledge, there are no prospective studies investigating artefacts on CMR scans in patients with LCP. We recently reported about a case of a patient with a LCP and an arc-shaped artefact at the RV apex [16]. As described by Klein-Wiele et al., pacing leads artefacts are smaller and do not usually interfere with myocardial structures which may be a potential advantage of transvenous cardiac pacemaker systems undergoing CMR imaging [19].

LCP are RV single-chamber pacemakers which are implanted by using a femoral percutaneous approach [12]. The next step of leadless technology will be dual-chamber pacing to treat patients with AV block [13]. Aurrichio et al. reported about feasibility, safety and short-term outcome of leadless ultrasound-based endocardial LV resychronization in patients with heart failure [22]. A study by Tjong FV et al. reported about leadless pacing combined with subcutaneous defibrillation therapy [23]. Therefore, the clinical use of these leadless cardiac devices will increase in the next years. Keller J et al. reported artefacts by the can of subcutaneous ICD affecting the LV [24]. This is consistent with our expierence in this patient undergoing CMR imaging that diagnostic evaluation of the LV is severely impaired by artefacts of the subcutaneous ICD.

CMR is an important non-invasive imaging tool to assess patients with cardiovascular diseases by evaluating myocardial function, wall motion abnormalities, viability, coronary perfusion, valves and tissue characterization [1]. Therefore, we were able to demonstrate that CMR is feasible in patients with LCP. Furthermore, the overall image quality was excellent to good and the artefact burden due to the LCP was comparable small and allowed a comprehensive evaluation of the cardiac structures and function, wall motion abnormalities and tissue characterization. However, mid anteroseptal, inferoseptal and apical septal myocardial segments of the LV were affected by artefacts due to the LCP which may impair or even exclude diagnostic evaluation of these segments, especially on perfusion and LGE images (Fig. 4). The artefact burden on CMR images could be significantly reduced by the use of 1.5 Tesla MRI scanners.

### Limitations of this study

Besides the limited number of patients this study has further limitations. This study enrolled only patients with Micra™ LCP and the described artefacts are specific for this LCP. Further studies are needed to evaluate the artefact burden of LCP from other manufactures. However, according to our data the size of the artefact by the LCP did not differ widely with only a small standard deviation on CMR images (0.99± 0.16 cm^2^). Therefore, we conclude that the localization of the artefact depends mainly on the site of implantation of the LCP which is usually at the apex of the RV or at the apical or mid right interventricular septum. The impaired RV function in 7 patients of our study population cannot be interpreted as a reduced RV function due to the LCP because we did not compare RV function before and after implantation of the LCP. Furthermore, we were not able to report on late gadolinum enhancement sequences as these sequences were not part of our CMR scan protocol. As reported by Klein-Wiele, late gadolinum enhancement sequences showed a lower artefact burden compared to bSSFP cine images [19].

## Conclusion

This prospective, single-center, observational study demonstrates that CMR imaging in patients with LCP implanted at least six prior to the CMR scan is feasible. Overall image quality was excellent to good in the majority of CMR images. Assessment of LV function and aortic, mitral and tricuspid valve as well as tissue characterization with T1 and T2 weighted imaging were feasible with 1.5 Tesla and 3 Tesla. However, mid anteroseptal, inferoseptal and apical septal myocardial segments of the LV were affected by artefacts of the LCP in the majority of patients which may impair or even exclude diagnostic evaluation of these segments. Artefact burden on CMR images may be reduced by the use of 1.5 Tesla scanners.

## Abbreviations

AHA: American Heart Association
bSSFP: Balanced steady-state free precession
CMR: Cardiovascular magnetic resonance
ECG: Electrocardiogram
FLASH: Fast low angle shot
GRE: Gradient echo
ICD: Implantable cardioverter defibrillators
LCP: Leadless cardiac pacemakers
LV: Left ventricle/left ventricular
MRI: Magnetic resonance imaging
RV: Right ventricle/right ventricular
TSE: Turbo spin echo
